# A prospective randomized comparison of the minimally invasive direct anterior and the transgluteal approach for primary total hip arthroplasty

**DOI:** 10.1186/s12891-018-2133-4

**Published:** 2018-07-19

**Authors:** Johannes C. Reichert, Eberhard von Rottkay, Franz Roth, Tim Renz, Johannes Hausmann, Julius Kranz, Lars Rackwitz, Ulrich Nöth, Maximilian Rudert

**Affiliations:** 1 Department of Orthopaedic Surgery, König-Ludwig-Haus, Center for Musculoskeletal Research, Julius-Maximilians-University, Brettreichstraße 11, 97074 Würzburg, Germany; 2 Department of Orthopaedic and Trauma Surgery, Evangelisches Waldkrankenhaus Spandau, Stadtrandstrasse 555, 13589 Berlin, Germany

**Keywords:** Total hip arthroplasty, Direct anterior approach, Minimally invasive, Transgluteal approach, Prospective study

## Abstract

**Background:**

The presented prospective randomized controlled single-centre study compares the clinical outcome up to 12 months after total hip arthroplasty using a minimally invasive single-incision direct anterior (DAA) and a direct transgluteal lateral approach.

**Methods:**

A total of 123 arthroplasties were evaluated utilizing the Harris Hip Score (HHS), the extra short musculoskeletal functional assessment questionnaire (XSFMA), the Short Form 36 (SF-36) health survey, a Stepwatch™ Activity Monitor (SAM), and a timed 25 m foot walk (T25-FW). Postoperative x-ray images after THA were reviewed to determine inclination and stem positioning.

**Results:**

At final follow-up, the XSFMA functional index scores were 10.3 (anterior) and 15.08 (lateral) while the bother index summed up to a score of 15.8 (anterior) and 21.66 (lateral) respectively, thus only differing significantly for the functional index (*p* = 0.040 and *p* = 0.056). The SF-36 physical component score (PCS) was 47.49 (anterior) and 42.91 (lateral) while the mental component score (MCS) summed up to 55.0 (anterior) and 56.23 (lateral) with a significant difference evident for the PCS (*p* = 0.017; *p* = 0.714). Patients undergoing THA through a DAA undertook a mean of 6402 cycles per day while those who had undergone THA through a transgluteal approach undertook a mean of 5340 cycles per day (*p* = 0.012). Furthermore, the obtained outcome for the T25-FW with 18.4 s (anterior) and 19.75 s (lateral) and the maximum walking distance (5932 m and 5125 m) differed significantly (*p* = 0.046 and *p* = 0.045). The average HHS showed no significant difference equaling 92.4 points in the anterior group and 91.43 in the lateral group (*p* = 0.477). The radiographic analysis revealed an average cup inclination of 38.6° (anterior) and 40.28° (lateral) without signs of migration.

**Conclusion:**

In summary, our outcomes show that after 1 year THA through the direct anterior approach results in a higher patient activity compared to THA utilizing a transgluteal lateral approach while no differences regarding hip function are evident.

**Trial registration:**

DRKS00014808 (German Clinical Trial Register DRKS); date of registration: 31.05.2018.

## Background

Total hip arthroplasty (THA) can generally be seen as a very successful procedure in the field of orthopaedics [1]. The demand for THA and especially minimally invasive surgeries (MIS) is continuously increasing [2] as successful THA results in pain relief and restored joint functionality allowing a higher degree of patient activity and therefore quality of life [3]. Minimally invasive THA is most commonly performed utilizing the direct anterior (DAA), the anterolateral and the posterior approach to the hip. MIS is associated with less blood loss [4], decreased perioperative pain levels, and faster recovery [3, 5]. However, there is still ample debate whether early functional outcomes differ using the DAA or a standard lateral approach [6].

Consequently, the primary aim of the present randomized controlled single-centre study was to prospectively compare the functional outcome, as measured by the HHS and SAM, between patients undergoing minimally invasive THA through a DAA with those undergoing THA through a conventional transgluteal approach. Secondary aims included comparison of the XSMFA (functional and bother index), a timed 25 m foot walk (T25-FW), and the short-form 36 questionnaire (SF-36 mental and physical).

## Methods

The present investigator sponsored, prospective randomized controlled single-centre study was approved by the ethics committee of the medical faculty of the University of Würzburg (approval number 72/11) and compares two different surgical techniques, the minimally invasive DAA as described by Rachbauer [7] and the lateral transgluteal approach according to Bauer et al. [8]. The DAA was introduced in our orthopaedic department in 2008 as a routine approach for THA. Prior to 2008 the transgluteal approach was predominately used. An a priori power analysis using G*Power 3 was performed to determine the necessary sample size *N* for a two-sample t test of independent variables given an α error probability of 0.05, a desired power level of 0.8 (1-β), and a medium effect size of 0.5. The required *N* was calculated to be 60 per group. With a power level of 0.9 a total of 81 patients would have been required in each group. The study was powered to detect a 500 step difference in measurements obtained with the SAM at a single time point assuming an average of 5500 steps/day and a standard deviation of 1000. A significance level of 5% was chosen.

The SAM (StepWatch Activity Monitor, Orthocare Innovations, 6405 218th St SW, Suite 100, Mountlake Terrace, WA 98043–2180, US) is an example of an accelerometer based activity monitor that has been used widely in different population groups. The SAM is small (75 × 50 × 20 mm) and lightweight (38 g) and is worn at the ankle. The monitor contains a custom sensor that uses a combination of acceleration, position, and timing to detect steps. Thus the outputs of the SAM are based on the amount, rate, and pattern of walking. The SAM is calibrated based on each individual’s height and gait pattern. Reported intraclass correlation coefficients (ICCs) for mean steps/day range from 0.86 to 0.99 representing an excellent test-retest reliability, and 95% limits of agreement of less than 40% [9].

Accounting for an expected drop out rate of 15% a number of 69 patients was planned to be recruited for each study arm. Between November 2011 and March 2014 148 consecutive patients were enrolled and allocated to the treatment groups by the principal investigator utilizing a computer generated block randomization list as described previously [10]. Block randomization is a commonly used technique to reduce bias and achieve balance in the allocation of participants to treatment arms. It increases the probability that each arm will contain an equal number of individuals by sequencing participant assignments by block. It was not possible to blind the patient for the allocated surgical technique, as the surgical incision site of the studied approaches was different.

**Fig. 1:**
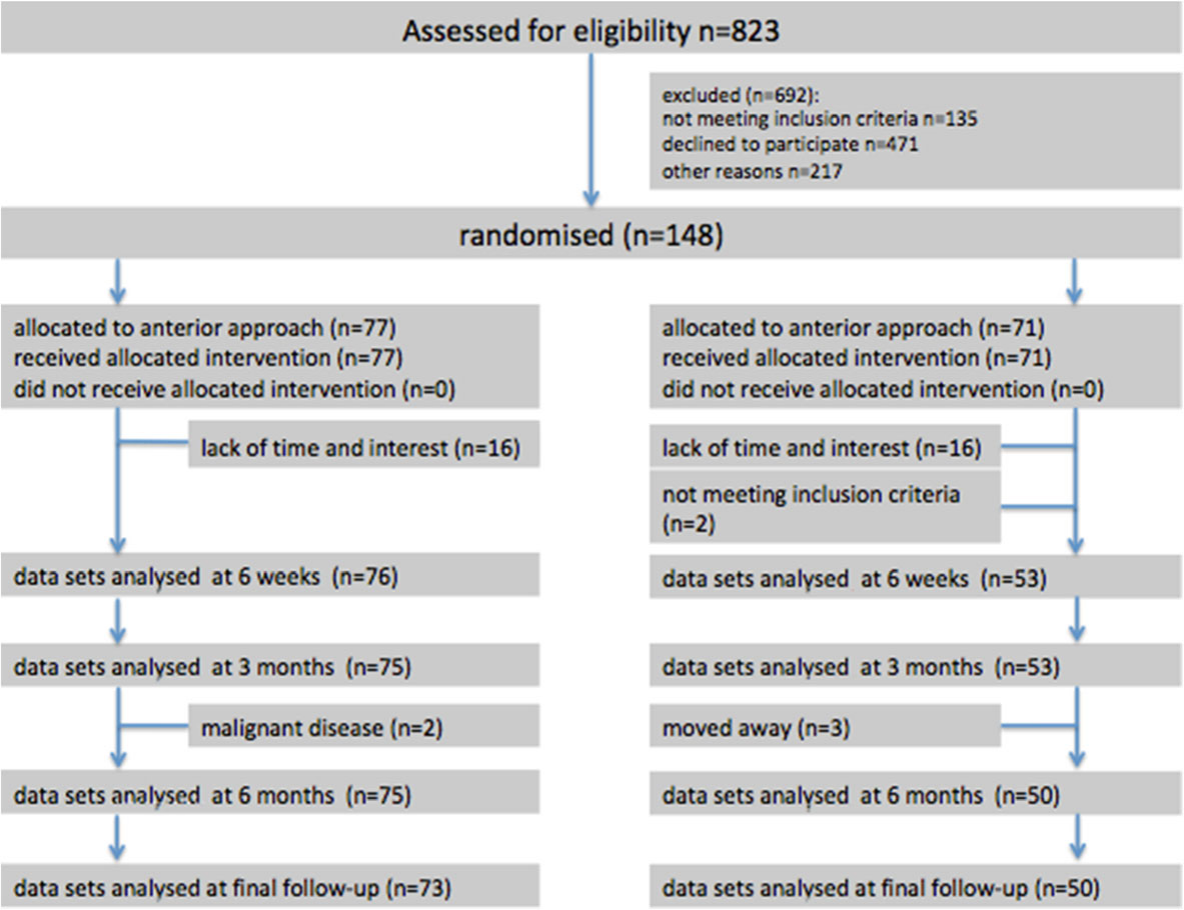
Flow diagram of the progress through the phases of the randomised trial

Four patients were lost to follow up in the anterior group whereas 21 individuals dropped out of the lateral group leaving 73 (anterior) and 50 (lateral) complete data sets for analysis respectively. The main reason for dropout was a lack of time, 3 patients had moved away, two patients were diagnosed with a malignant disease and two patients had to be excluded by hindsight not meeting the radiologic inclusion criteria (Fig. 1).

A high rate of eligible patients declined trial participation as they explicitly asked for THA through a minimally invasive direct anterior approach.

Eight experienced, fellowship-trained surgeons performed the surgeries (Table 1).

**Table 1:** Characteristics of evaluated patient collective preoperatively and at final follow-up

	Anterior		Lateral		
		Range		Range	*P* value
Preoperative					
Number of patients	77		71		
Age in years	63.2 (SD 8.2)	44-77	61.9 (SD 7.8)	50-78	0.193
Female	32 (42%)		32 (45%)		0.425
Body Mass Index (BMI)	28.1 (SD 3.7)	20.0-34.8	28.3 (SD 3.4)	20.9-42.2	0.869
Time of surgery	12/2011-3/2014		12/2011-3/2014		
Surgeons	8		8		
Final follow-up					
Number of patients	73		50		
Age in years	62.5 (SD 8.0)	44-77	62.2 (SD 8.5)	50-78	0.261
Female	32 (44%)		26 (52%)		0.195
Body Mass Index (BMI)	28.3 (SD 4.0)	20.0-34.8	28.7 (SD 3.2)	20.9-42.2	0.834

Patients with primary osteoarthritis scheduled for cemented or non-cemented THA were enrolled following defined inclusion and exclusion criteria.

Exclusion criteria were an age < 40 or > 80 years, a Body-Mass-Index (BMI) > 35 kg/m^2^; hip dysplasia or a congenital disorder of the hip, former osteotomies of hip, knee or pelvis; an impairment of the contralateral side or osteoarthritis of the ipsilateral knee, osteoporosis, degenerative spine disease, or a severe systemic disease (ASA-Score ≥ 4, malignant or cardiovascular disease).

The overall patient drop out and higher rate of patients lost to follow-up due to a lack of time and interest in the lateral group is considerable. However, both patient cohorts did not differ significantly after randomization regarding gender, age, BMI, educational and socioeconomic status, or any other specific patient characteristics thus contesting a general problem with randomization. As well, at baseline, completers did not differ from patients lost to follow-up.

The selected commercially available implants included Trilogy or Allofit cups (Trilogy® Acetabular Hip System; Allofit® Acetabular Cup System), the non-cemented M/L-Taper stem or the cemented M. E. Müller straight stem (all Zimmer). Overall, the anterior group included 4, the lateral group 5 cemented stems.

For all patients participating in the trial we applied established standardized treatment protocols, which included a multimodal pain management and rapid rehabilitation.

Patient examinations were carried out by a qualified physician 6 weeks prior to surgery and 6 weeks, 3, 6 and 12 months after surgery.

Activity was evaluated using the Stepwatch™ Activity Monitor (SAM) as the primary outcome measure (Orthocare Innovations, Oklahoma City, OK, USA) over a period of 7 days (preoperatively, 3 and 12 months after surgery) to calculate a daily average number of cycles [9].

Secondary outcome measures included the Harris hip score (HHS) [11] as an important clinical measure to evaluate hip function and the reciprocal extra short musculoskeletal functional assessment questionnaire (XSMFA functional and bother index, 16 items) [12]. The domains covered by the HHS are pain, function, absence of deformity and range of motion. The HHS includes ten items and has a maximum score of 100 representing the best possible outcome. Additional secondary measures were a timed 25 m foot walk (T25-FW), and a visual analogue scale (VAS, range 1–10) to further characterize patient activity. The individual patient health status was monitored using the short-form 36 questionnaire (SF-36 mental and physical component summaries) [13].

Conventional x-ray projections of the pelvis (anterior-posterior, frog leg view) were used to assess bony implant integration, cup inclination, and stem orientation (valgus, neutral or varus) as described previously by Johnston et al. [14]. Two independent observers performed the assessment. At the time of follow-up, clinical examination was performed for all patients, respectively.

Source data verification was conducted by the study team. After regular trial termination, acquired data was tested for normal distribution using both the Kolmogorov-Smirnov and Shapiro-Wilk test. In case of normally distributed dependent variables the two-tailed t-test was used for between group comparisons, the Mann-Whitney-U test for non-parametric data.

The non-parametric Friedman test was chosen to analyse repeated measures within the two patient groups. As a post hoc test, the Wilcoxon signed-rank test was used and a Bonferroni adjustment made. Statistical analysis was carried out using SPSS 19.0 (IBM, Ehningen, Germany), and *p*-values < 0.05 were considered significant.

## Results

In total, 77 arthroplasties were preformed through the minimally invasive DAA and 71 arthroplasties using the transgluteal lateral approach. The enrolment of more than 69 patients was allowed to possibly compensate for a drop out of more than nine patients per group.

Daily activity as determined by the StepWatch™ activity monitor resulted in an average of 4695 recorded load cycles per day preoperatively, 5992 (anterior) and 5239 (lateral) after 3 months, and 6402 and 5340 recorded load cycles after 1 year, respectively. The preoperatively recorded load cycles were pooled, as no statistical difference was determined between the groups (*p* = 0.489). Both 3 and 12 months after surgery, the patients operated using the direct anterior approach showed a significantly higher number of steps per day (*p* = 0.035 and *p* = 0.012) (Table 2). For the anterior group a significant increase was observed at final follow up (*p* < 0.001) compared to preoperative values and between month 3 and 12 (*p* = 0.047) while no significant change over time was seen for the lateral group (Table 3).

**Table 2:** Comparison of StepWatch activity monitor outcomes at each time point

StepWatch Approach	Mean (SD)		95% Cl		*P* value
	Anterior	Lateral	Anterior	Lateral	
Time points					
preoperative	4695 (1177)	4695 (1177)	4507-4887	4507-4887	n.a.
3 months	5992 (2170)	5239 (2309)	5501-6483	4617-5861	0.035^a^
12 months	6402 (2523)	5340 (1887)	5823-6981	4817-5863	0.012^a^

*SD* standard deviation, *Cl* confidence interval

^a^ Mann-Whitney-U test

**Table 3:** Within group comparison of StepWatch activity monitor outcomes over time

	Mean (SD)		*P* value		*P* value
StepWatch	Preoperative	3 months		12 months	
Approach					
Anterior	4695 (1177)	5992 (2170)	0.078^a^	6402 (2523)	0.047^a^
Lateral	4695 (1177)	5239 (2309)	1.00^a^	5340 (1887)	1.00^a^

*SD* standard deviation

^a^ Friedman test

The average HHS revealed very good clinical results. Compared to preoperative scores a significant increase was seen 6 weeks postoperatively (p≤0.001) and again between 6 weeks and 3 months (*p* = 0.039, Table 4) for both groups. No significant difference between the two surgical approaches was determined at any time point equalling 92.4 points in the anterior group and 91.4 in the lateral group 12 months after surgery (*p* = 0.477)(Table 5). Moreover, no statistically significant differences were found for the different domains of the HHS.

**Table 4:** Within group comparison of the HHS over time

	Mean (SD)		*P* value		*P* value		*P* value		*P* value
HHS	preoperative	6 weeks		3 months		6 months		12 months	
Approach									
Anterior	54.0 (14.2)	81.6 (12.1)	<0.001^a^	89.8 (9.3)	0.039^a^	90.3 (9.8)	1.000^a^	92.4 (8.6)	0.671^a^
Lateral	53.0 (15.7)	82.4 (12.0)	<0.001^a^	88.4 (9.9)	<0.001^a^	89.1 (10.0)	1.000^a^	91.4 (9.1)	0.526^a^

*SD* standard deviation

^a^ Friedman test

**Table 5:** Between group comparison of the HHS at each time point

HHS	Mean (SD)		95% Cl		*P* value
Approach	Anterior	Lateral	Anterior	Lateral	
Time points					
Preoperative	54.0 (14.2)	53.0 (15.7)	50.8-57.2	49.4-56.7	0.281^a^
6 weeks	81.6 (12.1)	82.4 (12.0)	78.3-83.7	78.8-85.2	0.068^a^
3 months	89.8 (9.3)	88.4 (9.9)	86.9-91.1	85.3-90.7	0.370^a^
6 months	90.3 (9.8)	89.1 (10.0)	87.8-92.2	86.2-91.8	0.556^a^
12 months	92.4 (8.6)	91.4 (9.1)	90.3-94.0	88.5-93.5	0.477^a^

*SD* standard deviation, *Cl* confidence interval

^a^ Mann-Whitney-U test

In comparison to the preoperative scores (35.2, SD 16.1 and 40.5, SD 16.0, *p* = 0.053) the average functional index of the reciprocal XSFMA decreased significantly 6 weeks postoperatively. Again a significant decrease was seen between 6 weeks and 3 months for both groups and between month 3 and 6 for the lateral group to result in a score of 10.3 (SD 13.0) 12 months after THA using the anterior approach and 15.1 (SD 16.3) using the lateral approach (Table 6). After 6 weeks as well as 3 and 12 months postoperatively patients who had received THA over an anterior approach achieved significantly better XSMFA functional indices when compared to patients of the lateral group. No significant difference between the groups was determined at baseline and the 6 months time point (Table 7).

**Table 6:** Within group comparison of the secondary outcome measures over time

	Mean (SD)		*P* value		*P* value		*P* value		*P* value
	Preoperative	6 weeks		3 months		6 months		12 months	
XSMFA fi									
Anterior	35.2 (16.1)	21.2 (14.2)	<0.001^a^	12.7 (12.5)	<0.001^a^	11.6 (12.1)	0.895^a^	10.3 (13.0)	1.000^a^
Lateral	40.5(16.0)	28.5(15.9)	<0.001^a^	18.8 (16.1)	<0.001^a^	15.8 (15.4)	0.037^a^	15.1 (16.3)	1.000^a^
XSMFA bi									
Anterior	48.7 (20.5)	26.6 (19.8)	<0.001^a^	19.8 (17.0)	0.005^a^	16.8 (15.8)	0.510^a^	15.8 (18.0)	1.000^a^
Lateral	53.0 (17.9)	33.0 (18.3)	<0.001^a^	33.0 (18.1)	0.021^a^	25.1 (17.9)	0.125^a^	21.7 (19.6)	1.000^a^
T25-FW									
Anterior	22.4 (5.2)	21.3(6.3)	0.743^a^	18.5 (3.7)	0.048^a^	18.3 (4.1)	1.000^a^	18.1 (3.4)	1.000^a^
Lateral	24.0 (3.9)	22.0 (4.2)	0.652^a^	19.4 (3.8)	0.049^a^	19.9 (5.5)	1.000^a^	19.8 (4.6)	1.000^a^
Activity VAS									
Anterior	5.0 (0.8)	6.9 (0.7)	<0.001^a^	7.3 (0.8)	1.000^a^	7.3 (0.7)	1.000^a^	7.5 (0.6)	1.000^a^
Lateral	4.9 (0.8)	6.8 (0.6)	<0.00^a^	6.9 (0.5)	1.000^a^	6.9 (0.7)	1.000^a^	7.0 (0.7)	1.000^a^
SF-36 PCS									
Anterior	27.4 (8.2)	39.1 (9.7)	<0.001^a^	44.6 (9.2)	<0.001^a^	46.0 (10.0)	1.000^a^	47.5 (9.9)	1.000^a^
Lateral	25.6 (8.7)	34.8 (9.8)	<0.001^a^	40.7 (10.1)	0.001^a^	42.7 (5.6)	0.277^a^	42.9 (11.9)	1.000^a^
SF-36 MCS									
Anterior	57.2 (8.5)	58.1 (8.7)	1,000^a^	56.0 (9.2)	1.000^a^	56.0 (10.0)	1.000^a^	55.0 (9.8)	1.000^a^
Lateral	56.3 (9.2)	59.3 (6.6)	0.931^a^	56.7 (8.3)	1.000^a^	55.8 (7.2)	1.000^a^	56.2 (6.9)	1.000^a^

*SD* standard deviation

^a^ Friedman test

**Table 7:** Between group comparison of the secondary outcome measures at each time point

Approach	Mean (SD)		95% Cl		*P* value
	Anterior	Lateral	Anterior	Lateral	
XSMFA functional index					
Preoperative	35.2 (16.1)	40.5(16.0)	31.4-38.6	36.3-43.7	0.053^a^
6 weeks	21.2 (14.2)	28.5(15.9)	17.8-24.2	23.7-32.3	0.026^a^
3 months	12.7(12.5)	18.8 (16.1)	9.2-14.8	13.7-22.3	0.023^a^
6 months	11.6(12.1)	15.8 (15.4)	8.3-13.7	10.7-19.3	0.094^a^
12 months	10.3(13.0)	15.1 (16.3)	7.3-13.3	10.5-19.5	0.040^a^
XSMFA bother index					
Preoperative	48.7 (20.5)	53.0 (17.9)	43.4-52.6	48.8-57.2	0.126^a^
6 weeks	26.6 (19.8)	33.0 (18.3)	21.6-30.5	28.1-37.9	0.055^a^
3 months	19.8 (17.0)	33.0 (18.1)	15.2-22.9	28.1-37.9	0.099^a^
6 months	16.8 (15.8)	25.1 (17.9)	12.4-19.6	20.0-30.0	0.149^a^
12 months	15.8 (18.0)	21.7 (19.6)	10.8-19.1	9.6-20.4	0.056^a^
T25-FW (s)					
Preoperative	22.4 (5.2)	24.0 (3.9)	20.8-23.2	23.1-24.9	0.193^a^
6 weeks	21.3(6.3)	22.0 (4.2)	19.6-22.4	20.0-23.1	0.385^a^
3 months	18.5 (3.7)	19.4 (3.8)	17.2-18.8	17.0-19.0	0.291^a^
6 months	18.3 (4.1)	19.9 (5.5)	17.1-18.9	17.5-20.5	0.040^a^
12 months	18.1 (3.4)	19.8 (4.6)	17.2-18.8	17.7-20.3	0.046^a^
Activity VAS					
Preoperative	5.0 (0.8)	4.9 (0.8)	4.8-5.2	3.8-4.2	0.461^a^
6 weeks	6.9 (0.7)	6.8 (0.6)	5.8-6.2	5.8-6.2	0.031^a^
3 months	7.3 (0.8)	6.9 (0.5)	6.8-7.2	5.9-6.1	0.080^a^
6 months	7.3 (0.7)	6.9 (0.7)	6.8-7.2	6.7-7.1	0.223^a^
12 months	7.5 (0.6)	7.0 (0.7)	6.9-7.1	6.8-7.2	<0.001^a^
Walking distance (m)					
12 months	6435 (4260)	5125 (3868)	5458-7412	4053-6197	0.045^a^
SF-36 PCS					
Preoperative	27.4 (8.2)	25.6 (8.7)	25.5-28.8	23.0-27.0	0.152^a^
6 weeks	39.1 (9.7)	34.8 (9.8)	36,8-41,2	31.4-36.6	0.004^a^
3 months	44.6 (9.2)	40.7 (10.1)	41.9-46.1	37.3-42.7	0.031^a^
6 months	46.0 (10.0)	42.7 (5.6)	43.7-48.3	40.5-43.6	0.042^a^
12 months	47.5 (9.9)	42.9 (11.9)	44.7-49.3	40.1-43.9	0.017^a^
SF-36 MCS					
Preoperative	57.2 (8.5)	56.3 (9.2)	55.1-58.9	53.9-58.1	0.405^a^
6 weeks	58.1 (8.7)	59.3 (6.6)	56.0-60.0	57.2-60.8	0.465^a^
3 months	56.0 (9.2)	56.7 (8.3)	53.9-58.1	53.8-58.2	0.774^a^
6 months	56.0 (10.0)	55.8 (7.2)	53.7-58.3	53.0-57.0	0.670^a^
12 months	55.0 (9.8)	56.2 (6.9)	52.8-57.3	54.1-57.9	0.714^a^

*SD* standard deviation, *Cl* confidence interval

^a^ Mann-Whitney-U test

For the obtained bother indices no significant difference was found between the groups at any time point (Table 7). The preoperative bother index equalled 48.7 (SD 20.5, anterior) and 53.0 (SD 17.9, lateral) and significantly improved to 15.8 (SD 18.0, anterior) and 21.66 (SD 19.55, lateral) after 12 months. The increase after 6 weeks compared to preoperative values was significant for both groups (*p* < 0.001), furthermore a significant increase was determined between 6 weeks and 3 months (*p* = 0.005 anterior; *p* = 0.021 lateral) (Table 6).

For the timed 25 m foot walk (T25-FW) the results after 6 weeks were significantly better compared to preoperative measurements in both groups (*p* = 0.038, anterior; *p* = 0.041, lateral), furthermore a significant increase was determined between 6 weeks and 3 months (*p* = 0.043 anterior; *p* = 0.045 lateral) (Table 6). The between group comparison showed no significant difference preoperatively and 6 and 12 weeks after intervention. However, 6 and 12 months after surgery, the patients in the anterior group showed significantly better results averaging 18.3 s (SD 4.1, anterior) compared to 19.9 s (SD 5.53, lateral, *p* = 0.040) and 18.1 s (SD 3.4, anterior) versus 19.8 (SD 4.6, lateral, *p* = 0.046) (Table 7).

Activity levels as assessed by the patients on a visual analogue scale (range 1–10) showed no significant difference preoperatively with 5.0 (SD 1.85, anterior) and 4.9 (SD 1.80, lateral) points (*p* = 0.577). Similar results were obtained after 6 weeks (6.9, SD 1.92 and 6.8, SD 2.90; *p* = 0.75). After 3 months, however, the patients of the anterior group described significantly higher activity levels (7.3, SD 1.65 and 6.8 SD 1.35; *p* = 0.032), which were confirmed at the final follow-up (7.5, SD 1.82 and 7.0, SD 1.57, *p* = 0.016) (Table 7). For both cohorts, the outcome 6 weeks after surgery was significantly better compared to preoperative measurements (each *p* < 0.001) (Table 6).

In addition the patients of the anterior group reported a significantly higher maximum walking distance before the onset of pain with an average of 6435 m (SD 4260) compared to 5125 m (SD 3868, *p* = 0.045, Table 7) at final follow-up.

The average scores obtained in the physical component of the SF-36 (PCS) were calculated to be 27.5 (SD 8.5, anterior) and 26.7 (SD 8.6, lateral) points preoperatively, significantly increasing to 39.7 (SD 9.7) and 37.8 (SD 9.99) 6 weeks after surgery (*p* < 0.001). Between weeks 6 and 12 after surgery again a significant increase to 44.6 (SD 9.16) and 43.1 (SD 9.72) was determined (*p* < 0.001, *p* = 0.001). The score after 6 months summed up to 46.0 (SD 10.0) and 44.8 (SD 9.95) finally resulting in 47.5 (SD 9.9) and 45.7 (SD 10.9) points 12 months postoperatively. The increase between the 3 and 12 month outcome was again statistically significant (*p* = 0.041 and *p* = 0.043) (Table 6). At each postoperative time point the patients in the anterior group achieved significantly higher scores (*p* = 0.04, *p* = 0.031, *p* = 0.042 and *p* = 0.017) (Table 7). The mental component (MCS) summed up to a score of 57.2 (SD 8.4, anterior) and 56.3 (SD 9.2, lateral) before surgery. The MCS did not change significantly over time (Tables 6 and 7).

Generally postoperative pain reduction was assessed to be good to very good. Pain intensity measurements revealed that as early as 6 months after surgery 94% (anterior) and 96% (lateral) of all patients were free of pain sensations.

The radiographic analysis revealed an average cup inclination of 38.6° (SD 5.7) in the anterior group compared to 40.3° (SD 6.2) in the lateral approach group (Table 8). None of the cups in either group presented with evidence of migration after 1 year.

**Table 8:** Radiographic analysis of cup placement; surgery related complications

Measure	Anterior	Lateral
Mean inclination (°)	38.6 (SD 5.7)	40.28 (SD 6.2)
Exchange of parts	1 (aseptic loosening, 1.4%)	0
Persistent lateral femoral cutaneous nerve paraesthesia	3 (4.1%)	0
Joint dislocation	0	1
Leg length discrepancy >1cm	2 (2.7%)	3 (6%)
Trendelenburg	1 (1.4%)	1 (2%)

Stems positioning was assessed to be neutral in 92.5% (anterior) and 94% (lateral) of all cases while 5.5% (anterior) and 4% (lateral) were graded varus. A valgus stem orientation was found in 2% of cases in both groups.

In case of non-cemented THA, all implanted cups and stems showed radiographic evidence of osseointegration, regardless of the approach used.

Peri- and postoperative complications with THA trough the DAA were as follows: We observed three cases of lateral femoral cutaneous nerve (LFCN) palsy and performed one revision surgery due to aseptic loosening. All LFCN deficits had resolved spontaneously at final follow-up. Furthermore, a positive Trendelenburg sign indicative of gluteal insufficiency was observed in one patient. In addition, two leg length discrepancies > 1 cm occurred. These were clinically relevant and treated with orthopaedic insoles. In the lateral group one joint dislocation occurred, which was treated conservatively, moreover three leg length discrepancies compensated for with insoles, and one gluteal insufficiency.

## Discussion

The demand for minimally invasive hip arthroplasty is continuously increasing [15]. Literature provides evidence for a decreased intra-operative blood loss, lower postoperative pain levels and a shorter recovery time in case of minimally invasive surgery [3–5]. The functional mid-term and long-term outcomes after THA using minimally invasive techniques are comparable with those of THA via standard approaches [16, 17]. The number of randomized trials to underline these findings, however, is still scarce. Consequently, we initiated a prospective randomized study comparing the minimally invasive DAA and standard transgluteal approach.

This study compared outcomes in 73 patients undergoing minimally invasive THA through a DAA with those in 50 patients undergoing THA through a direct lateral approach showed comparable or even superior functional outcomes and activity levels of the anterior approach group as evaluated by the HHS, XSFMA, SF-36, SAM, and T25-FW. The results obtained for both groups are within the expected range and similar to those reported in previous studies [18–20].

Functionality as represented by the HHS did not reveal a significant difference while obtained XSMFA functional indices differed significantly. Overall, our study results are in line with previous reports on minimal-invasive hip replacement [21, 22]. The differing outcome comparing the HHS and XSMFA, however, may find its reason in the different weightings of these scores. The subdivisions of the HHS are pain, functionality and deformities while the XSMFA is composed of two indices (functionality and bother) only. Moreover, the discriminative ability oft the HHS is impaired having a limited number of challenging items thus resulting in a ceiling effect [23]. Consequently, the patients who underwent THA by direct anterior MIS show a better functionality while the level of impairment is similar as in the lateral group 1 year after surgery. This is also reflected in the results of the SF-36 physical component score.

The StepWatch™ Activity Monitor represents an objective method of taking measurement. The results obtained with the SAM differed significantly between both cohorts. A comparison of these outcomes with previous literature reports proves difficult as a variety of devices were applied to record patient activity in the past. These include mechanical pedometers or traditional accelerometer-based step counting modalities. Furthermore, the periods of patient observation were greatly varying [24]. Nevertheless, the obtained study results correspond well with previously published patient activity levels after THA [25–27]. These outcomes are corroborated by the T25-FW measurements and the determined maximum walking distance at final follow up 12 months after surgery (Table 2).

We observed a higher incidence of varus positions in the DAA group, which was, however, not statistically significant but is a generally known phenomenon [28].

Moreover, a palsy rate of the lateral femoral cutaneous nerve (LCFN) of 4.1% was seen using the DAA although even higher incidences are described (14.8%) [29, 30]. The LCFN is a sensory nerve and supplies the anterior and lateral thigh. Its anatomic location varies greatly [31]. As a result, the LCFN is generally prone to injury utilizing a DAA. Persistent neuralgias were, however, not observed.

Joint dislocation after THA remains an unsolved problem. With the anterior approach rates of approximately 0.6–1.6% [32] are to be expected. In our study, one patient was diagnosed with a dislocated hip in the lateral group while no dislocation occurred in the DAA group.

Notably, one main advantage of the DAA over the lateral approach is that the gluteus tendons/muscles are left unharmed [33, 34]. With lateral approaches, on the other hand, gluteal insufficiency is observed regularly [35]. It was, however, observed in one study patient in each group.

Our study has several limitations. Only 21.5% of individuals found to be eligible for this study were enrolled. This could potentially lead to sampling bias. Due to patient loss to follow-up the possibility of an inadequate sample size must be considered, especially in regards to the lateral group. Another factor to criticise might be the strict set of exclusion criteria defined what might have influenced the results of both study groups.

Despite the above-mentioned limitations, we conclude that the minimally invasive DAA leads to similar or even superior clinical short and mid-term outcomes when compared to the lateral transgluteal approach.

## Conclusions

THA in general significantly increases patients’ activity, functionality and general physical health. After 1 year our outcomes show that THA through the direct anterior approach results in a higher patient activity compared to THA utilizing a transgluteal lateral approach while no differences regarding hip function as represented by the HHS are evident.

## Abbreviations

ASA: American Society of Anesthesiologists
DAA: Direct anterior approach
HHS: Harris Hip Score
LFCN: Lateral femoral cutaneous nerve
MCS: Mental component score
PCS: Physical component score
SAM: Stepwatch™ Activity Monitor
SF-36: Short Form 36
T25-FW: Timed 25 m foot walk
THA: Total hip arthroplasty
VAS: Visual analogue scale
XSMFA: The extra short musculoskeletal functional assessment questionnaire
